# The surprising negative correlation of gene length and optimal codon use - disentangling translational selection from GC-biased gene conversion in yeast

**DOI:** 10.1186/1471-2148-11-93

**Published:** 2011-04-11

**Authors:** Nina Stoletzki

**Affiliations:** 1 Ludwig-Maximilan Universität, Biocenter, Grosshadernerstr. 2, D-82152 Planegg-Martinsried, Germany; 2 Centre for the Study of Evolution, School of Life Sciences, University of Sussex, Brighton BN1 9QG, UK; 3 Current Address: Division of Genetics, Brigham and Women's Hospital, Harvard Medical School, 77, Louis Pasteur Avenue, NRB, Boston, MA, USA

**Keywords:** GC-biased gene conversion, optimal codon use, cause of correlation

## Abstract

**Background:**

Surprisingly, in several multi-cellular eukaryotes optimal codon use correlates negatively with gene length. This contrasts with the expectation under selection for translational accuracy. While suggested explanations focus on variation in strength and efficiency of translational selection, it has rarely been noticed that the negative correlation is reported only in organisms whose optimal codons are biased towards codons that end with G or C (-GC). This raises the question whether forces that affect base composition - such as GC-biased gene conversion - contribute to the negative correlation between optimal codon use and gene length.

**Results:**

Yeast is a good organism to study this as equal numbers of optimal codons end in -GC and -AT and one may hence compare frequencies of optimal GC- with optimal AT-ending codons to disentangle the forces. Results of this study demonstrate in yeast frequencies of GC-ending (optimal AND non-optimal) codons decrease with gene length and increase with recombination. A decrease of GC-ending codons along genes contributes to the negative correlation with gene length. Correlations with recombination and gene expression differentiate between GC-ending and optimal codons, and also substitution patterns support effects of GC-biased gene conversion.

**Conclusion:**

While the general effect of GC-biased gene conversion is well known, the negative correlation of optimal codon use with gene length has not been considered in this context before. Initiation of gene conversion events in promoter regions and the presence of a gene conversion gradient most likely explain the observed decrease of GC-ending codons with gene length and gene position.

## Background

Translational selection on synonymous codon use is indicated if frequencies of preferentially used, 'optimal', codons increase with expression level and correspond to the most abundant tRNA or to the tRNA with which they form the strongest binding - for several organisms, this seems to be the case (see for reviews [1-4]). Additional support for a beneficial role of certain 'optimal' codons in translation comes from laboratory studies [5-8]. Translational selection may act to maximise the speed of elongation, minimize the costs of proofreading or maximise the accuracy of translation [1], and depending on the selective target, one can test further distinct predictions. Under selection for translational accuracy we expect for example: (i) selection against translational errors to relate to the error's costs. As costs of an erroneous protein should accrue with each added amino acid during protein synthesis, one may expect long genes to experience higher optimal codon use than short genes [9]. Supporting selection for translational accuracy, in *E. coli *and yeast, relative optimal codon use indeed increases with gene length [9-12]. (ii) We also expect selection against translational errors to relate to the error's functional effect: translational errors for some amino acids may have no functional effects, while for other amino acids, translational errors render a protein non-functional. The latter should be under stronger selection for translational accuracy. As Akashi [13] points out, the functional importance of amino acid site may be approximated by its evolutionary conservation. Under translational selection for accuracy one may hence expect higher optimal codon frequencies at conserved than at non-conserved amino acid sites. This is indeed the case in *D. melanogaster*, *C. elegans*, *E. coli *[12-14]; a recent study [15] indicates this pattern may also apply to mouse and human using a modified measure of optimal codons.

However, surprisingly, in *D. melanogaster*, *C. elegans*, *A. thaliana*, and humans, optimal codon use decreases with gene length, thereby opposing the prediction under selection for translational accuracy [16-18]. This decrease is particularly surprising for species, in which selection for translational accuracy is indicated by the aforementioned higher optimal codon use at conserved amino acid sites. The explanation may be that the negative correlation between optimal codon use and gene length simply is a side effect: highly expressed genes with high optimal codon use tend to be short, possibly to be more economic [19]. Yet, while control of expression level affects the correlation of optimal codon use with gene length in yeast, causing a change from negative to positive [10,11], in *D. melanogaster*, *C. elegans*, *A. thaliana *or humans, the negative correlation of optimal codon use with gene length does not seem to be due to a correlation of gene length with expression level only [11,17,18,20,21].

Two explanations for the negative correlation have been proposed, both of which are based on translational selection. First, under selection for translational efficiency, selection for optimal codons may decrease with gene length due to the decrease in the relative fitness effect per optimal codon [16]. The second hypothesis invokes Hill-Robertson interference [22], which considers the reduction in selective efficacy due to linkage among sites: weakly or strongly selected sites that evolve adaptively or under constraints may affect evolutionary dynamics of linked sites. As Comeron et al. [16] suggest selection efficacy on optimal codons may decrease with gene length, as long genes with higher numbers of potentially interfering sites may experience a stronger Hill-Robertson effect. The Hill-Robertson effect has been considered for various effects on synonymous codons [e.g. [23-29]]. As recombination breaks down linkage, the observed positive correlation of optimal codon use with recombination rate was taken as support for the Hill-Robertson effect reducing the efficacy of translational selection on optimal codons [30-32].

Yet, optimal codons in several metazoans, such as the ones for which the negative correlation was first reported for, i.e. *D. melanogaster*, *C. elegans *and *A. thaliana*, but also for humans are mostly ending with G or C (-GC) [see codon tables in [17,18]], and compositionally biased mutation or repair processes may indirectly affect optimal codon use. Recombination-dependent repair (gene conversion) is indeed biased towards -GC in many organisms including yeast, mice, humans and Drosophila [20,33-36], and hence may be the potential force. Effects of GC-biased gene conversion will be most obvious at sites that evolve neutrally or under weak selection, and the substitution patterns it leaves resembles that of directional selection [see for review [36]]. GC-biased gene conversion has been indicated to affect optimal codon use before: optimal codon frequencies increase with recombination rate, a patterns consistent with population genetic predictions under translational selection on optimal codons [30,31]. However, in *D. melanogaster *and *C. elegans *not only optimal codon frequencies increase with recombination, but also non-optimal ones, as long as they end with -GC [20,37]. The positive correlation of *non-optimal *GC-ending codon frequencies with recombination indicates the observed positive correlation *optimal (GC-ending) *codons - that was taken as evidence for reduced efficacy of translational selection due to Hill-Robertson interference - is likely affected by compositionally biased processes such GC-biased gene conversion [20,37]. Whether or not GC-biased gene conversion or Hill-Robertson effects the positive correlation between optimal codons and recombination attracted controversy [see for example [20,37] versus [31,32]], but with respect to the observed negative correlation of optimal codon use with gene length, GC-biased gene conversion has never been considered.

The negative correlation of optimal codon use with gene length is found in organisms whose optimal codons are biased towards GC-ending ones, and may hence be caused by forces acting on optimal codons or on base composition. As translational selection affects optimal codons, while a compositional bias like gene conversion affects GC-ending codons, one may disentangle the effects by looking at optimal and non-optimal GC- and AT-ending codons separately. *Saccharomyces cerevisiae *is a good organism to disentangle the two forces because translationally optimal codons are not biased towards GC-ending ones as in the above mentionned organisms. Furthermore, translational selection and GC-biased gene conversion are comparably well-studied and supported in *S. cerevisiae *[e.g. [34,38,39]].

Results of this study demonstrate in *S. cerevisiae *the frequency of GC-ending (*optimal *AND *non-optimal) *codons decreases with gene length and increases with recombination. Also a decrease of GC-ending codons along genes is indicated. This distinction between AT- and GC-ending codons cannot be explained by variation in strength and efficiency of translational selection, while GC-biased gene conversion may explain the observation. Substitutions at four-fold degenerated sites differ between AT->GC and GC->AT changes, further supporting an effect of GC-biased gene conversion. Initiation of gene conversion events in promoter regions and the presence of a gene conversion gradient most likely explain the observed decrease of GC-ending codons with gene length and gene position.

## Results

### Difference between optimal codons depending on whether they end with -AT or -GC

To disentangle forces affecting base compositions (such as GC-biased gene conversion) from translational selection on optimal codons, one can compare optimal GC-ending and optimal AT-ending codons. As expected under translational selection, all optimal codons independent of their third nucleotide increase with gene expression (Table 1). As expected under GC-biased gene conversion, only the frequency of GC-ending optimal codons increases with recombination, AT-ending optimal codons however tend to be negatively or not significantly correlated with recombination (Table 1). Crucially with respect to gene length, only the relative frequencies of AT-ending optimal codons increase with gene length as one may expect under selection for translational accuracy. In contrast, the relative frequencies of GC-ending optimal codons decrease with gene length. This is true for individual amino acids, as well as for groups of amino acids with either AT-or with GC-ending optimal codons (F_OP_AT, and F_OP_GC respectively; Table 1). This distinction between optimal AT- and optimal GC-ending codons indicates an effect related to base composition.

**Table 1:** Difference of optimal codons depending on whether they end with -GC or -AT.

Amino acid	Gene length	Expression	Recombination
**F**_**OP**_**AT: F**_**OP **_**for aminoacids with AT-ending optimal codons**			

**Ala** N = 53719 codons	+0.0229NS	+0.2546***	Co:-0.0656** nCo:-0.0217NS spo11:-0.0856*** dmc1:-0.0540* mre11_0_:+0.0147NS mre11_6_:-0.0659**

**Arg** N = 43139 codons	+0.0002NS	+0.4301***	Co:+0.0293NS nCo:+0.0265NS spo11:+0.0297NS dmc1:+0.0488NS mre11_0_:+0.0375NS mre11_6_:-0.0037NS

**Cys** N = 13614 codons	+0.0535*	+0.2293***	Co:-0.0162NS nCo:-0.0239NS spo11:-0.0229NS dmc1:+0.0030NS mre11_0_:+0.0172NS mre11_6_:-0.0158NS

**Gln** N = 38581 codons	+0.0603*	+0.2076***	Co:-0.0777** nCo:-0.0546* spo11:-0.0495NS dmc1:-0.0873*** mre11_0_:-0.0532NS mre11_6_:-0.0565*

**Glu** N = 65707 codons	+0.1396***	+0.1663***	Co:-0.0658** nCo:-0.0573* spo11:-0.0796** dmc1:-0.1154*** mre11_0_:-0.0252NS mre11_6_:-0.0822**

**Gly** N = 49861 codons	+0.0470NS	+0.4385***	Co:+0.0241S nCo:+0.0230NS spo11:+0.0298NS dmc1:+0.0406NS mre11_0_:+0.0831** mre11_6_:-0.0037NS

**Pro** N = 43069 codons	+0.0304NS	+0.3114***	Co:-0.0509* nCo:-0.0292NS spo11:+0.0105NS dmc1:-0.0481NS mre11_0_:+0.0116NS mre11_6_:-0.0348NS

**Grouped** N = 307690 codons R^2 ^= 0.300*** P(GL)***,P(Expr)***, P(dmc1)***P(mre11_0_)**	+0.1271***	+0.3732***	Co:-0.0867** mCo:-0.0556* spo11:-0.0086*** dmc1:-0.1643*** mre11_0_:-0.0601* mre11_6_:-0.1074***

**F**_**OP**_**GC**: F_OP _for aminoacids with GC-ending optimal codons			

Leu N = 96687 codons	-0.1031***	+0.3690***	Co:+0.1828*** nCo:+0.1451*** spo11:+0.2015*** dmc1:+0.2526*** mre11_0_:+0.1206*** mre11_6_:+0.1344***

**Asn** N = 60930 codons	-0.1588***	+0.2880***	Co:+0.2101*** nCo:+0.2000*** spo11:+0.2687*** dmc1:+0.3655*** mre11_0_:+0.1991*** mre11_6_:+0.1866***

**Asp** N = 60058 codons	-0.1995***	+0.1485***	Co:+0.1976*** nCo:+0.1500*** spo11:+0.2602*** dmc1:+0.3561*** mre11_0_:+0.1437*** mre11_6_:+0.1997***

**Tyr** N = 32887 codons	-0.1850***	+0.2240***	Co:+0.1718*** nCo:+0.1401*** spo11:+0.2499*** dmc1:+0.3186*** mre11_0_:+0.1834*** mre11_6_:+0.1704***

**Phe** N = 44129 codons	-0.1103***	+0.2729***	Co:+0.2689*** nCo:+0.1612*** spo11:+0.2628*** dmc1:+0.3413*** mre11_0_:+0.1933*** mre11_6_:+0.1772***

**Lys** N = 72564 codons	-0.2207***	+0.3210***	Co:+0.2248*** nCo:+0.1475*** spo11:+0.2843*** dmc1:+0.3632*** mre11_0_:+0.1471*** mre11_6_:+0.2043***

**His** N = 21966 codons	-0.1010***	+0.1060***	Co:+0.1193*** nCo:+0.1132*** spo11:+0.1623*** dmc1:+0.2096*** mre11_0_:+0.1086*** mre11_6_:+0.1357***

**Grouped** N = 389221 codons R^2 ^= 0.424, P(GL)***,P(Expr)*** P(dmc1, mre11_0_)***	-0.2604***	+0.4005***	Co:+0.2935*** nCo:+0.2291*** spo11:+0.3667*** dmc1:+0.5090*** mre11_0_:+0.2341*** mre11_6_:+0.2724***

Spearman Rank Correlations between optimal codon use, gene length, expression, and various recombination measures for amino acids and groups of amino acids with either AT- or with GC-ending optimal codons. 1554 genes for which all amino acids were present at least four times were used. Presented for grouped variables are also MR results of variables whose log-transformation did not grossly deviate from a normal distribution. *P < 0.05, **P < 0.01,***P < 0.001, NS = not significant.

Interrelated variables such as expression level may affect correlations between codon use and gene length. To control for all possible interrelated variables, and especially for noisy variables such as expression level is not easy [40,41]. However, as the same genes were used for comparing AT- and GC-ending optimal codons, a control of other variables is not necessary to highlight their difference. AT- and GC-ending optimal codons are sampled from the same distributions of other potentially affecting variables such as expression level. The effect of gene expression (and other variables) should hence affect them both similarly. The major difference between AT- and GC-ending optimal codons can hence be demonstrated by simple Spearman rank correlation analyses: expression level should affect all (AT- and GC-ending) optimal codons similarly, while clearly, GC-ending codons differ from AT-ending codons in their correlation with gene length and with recombination (Table 1). The opposing correlation of AT- and GC-ending optimal codons with gene length and recombination indicates another force unrelated to translational selection is acting.

Multiple regression (MR) analysis between log-transformed F_OP_GC or F_OP_AT estimates, gene length, expression and two different measures of recombination confirms independent effects of expression, recombination and gene length (Table 1). Please be aware however that both, recombination and expression measures will be noisy and as Plotkin and Fraser [40] highlight, one should not compare the explanatory power of predictors with standard regression techniques when the predictors contain different amounts of measurement noise.

### Effect of base composition independent of translational selection

For amino acids with at least two optimal or with at least two non-optimal codons, one can estimate the relative GC-content of either optimal or at non-optimal codons separately. For amino acids which have two optimal codons, I estimate the frequency of GC-ending optimal codons relative to all optimal codons (F_GC_Opt); for amino acids which have at least two non-optimal codons, I estimate the frequency of GC-ending non-optimal codons relative to all non-optimal (F_GC_NonOpt). To look at optimal and non-optimal codons separately controls for translational selection. The analysis supports an effect of base composition: relative frequencies of GC-ending codons (F_GC_Opt and F_GC_Non-opt) consistently decrease with gene length and increase with recombination (Table 2). For all but Arg, the correlations are significant per amino acid; when grouped across respective amino acids, the decrease is significant for both F_GC_Opt and F_GC_Non-opt (Table 2).

**Table 2:** Effect of base composition independent of translational selection.

Amino acid	Gene length	Expression	Recombination
**F**_**GC**_**opt: Frequency of GC-ending optimal codons, n = 1506 genes**			

**Ile** N = 47505 codons	-0.1567***	+0.1026***	Co:+0.2280*** nCo:+0.2027*** spo11:+0.2754*** dmc1:+0.3490*** mre11_0_:+0.1663*** mre11_6_:+0.2185***

**Ser** N = 36478 codons	-0.1054***	+0.0354NS	Co:+0.1602*** nCo:+0.0926*** spo11:+0.1722*** dmc1:+0.2429*** mre11_0_:+0.1185*** mre11_6_:+0.1602***

**Thr** N = 30830 codons	-0.0899**	+0.0837**	Co: = 0.2019*** nCo:+0.1365*** spo11:+0.2252*** dmc1:+0.2827*** mre11_0_:+0.0958** mre11_6_:+0.1302***

**Val** N = 32710 codons	-0.1544***	+0.0954***	Co:+0.2036*** nCo:+0.1433*** spo11:+0.2371*** dmc1:+0.3038*** mre11_0_:+0.1336*** mre11_6_:+0.1802***

**Grouped** N = 261651 codons R^2 ^= 0.194*** P(GL)***,P(Expr)** P(dmc1, mre11_0_)***	-0.2153***	+0.1304***	Co:+0.3181*** nCo:+0.2274*** spo11:+0.3667*** dmc1:+0.4767*** mre11_0_:+0.2293*** mre11_6_:+0.2769***

**F**_**GC**_**non-opt: Frequency of GC-ending non-optimal codons, n = 1276 genes**			

**Ala** N = 30071 codons	-0.0855**	+0.1573***	Co:+0.1592*** nCo:+0.1124*** spo11:+0.2234*** dmc1:+0.2520*** mre11_0_:+0.1173*** mre11_6_:+0.1586***

**Arg** N = 15296 codons	-0.0152NS	+0.2595***	Co:+0.0435NS nCo:+0.0651* spo11:+0.1235*** dmc1:+0.1255*** mre11_0_:+0.0714* mre11_6_:+0.0893**

**Gly** N = 23663 codons	-0.0689*	+0.1643***	Co:+0.1707*** nCo:+0.1559*** spo11:+0.2027*** dmc1:+0.2850*** mre11_0_:+0.1697*** mre11_6_:+0.1397***

**Leu** N = 62345 codons	-0.1961***	-0.0819**	Co:+0.2238*** nCo:+0.1806*** spo11:+0.2578*** dmc1:+0.3524*** mre11_0_:+0.1106*** mre11_6_:+0.1979***

**Pro** N = 23167 codons	-0.1141***	-0.1121***	Co:+0.1576*** nCo:+0.1333*** spo11:+0.1384*** dmc1:+0.2066*** mre11_0_:+0.0792** mre11_6_:+0.1310***

**Thr** N = 22921 codons	-0.0736**	-0.0469NS	Co:+0.1404*** nCo:+0.1070*** spo11:+0.1571*** dmc1:+0.1914*** mre11_0_:+0.0906** mre11_6_:+0.0981***

**Ser** N = 45898 codons	-0.1153***	-0.0056NS	Co:+0.1943*** nCo:+0.1194*** spo11:+0.2200*** dmc1:+0.2990*** mre11_0_:+0.1669*** mre11_6_:+0.1775***

**Val** N = 20558 codons	-0.0599*	+0.1127***	Co:+0.2433*** nCo:+0.1623*** spo11:+0.1956*** dmc1:+0.2672*** mre11_0_:+0.1468*** mre11_6_:+0.1747***

**Grouped** N = 341847 codons R^2 ^= 0.727*** P(GL)***,P(Expr)*** P(dmc1)***, P(mre11_0_)NS	-0.2858***	+0.0820**	Co:+0.3194*** nCo:+0.2443*** spo11:+0.3526*** dmc1:+ 0.5357*** mre11_0_:+ 0.2620*** mre11_6_:+0.2978***

Spearman Rank Correlations between the frequency of GC-ending optimal or non-optimal codons with gene length, expression, and various recombination measures. For each amino acid, only genes were used for which at least 4 optimal or 4 non-optimal codons were present respectively. Presented for grouped variables are also MR results of variables whose log-transformation did not grossly deviate from a normal distribution. *P < 0.05, **P < 0.01,***P < 0.001, NS = not significant.

Notably, the analysis also indicates expression may affect the frequencies of GC-ending codons: controlling for translational selection, GC-ending codons, F_GC_Opt and F_GC_Non-opt, increase with expression level. This may be a side effect: first, in yeast, recombination and gene expression seem coupled [42] and secondly, highly expressed genes tend to be short [19]. However, it may also reveal some selective force: GC-ending codons affect for example thermodynamic stability and hence mRNA secondary structures which may be under selection [43-46] (see also Discussion). MR analysis between log-transformed variables supports independent effects of gene length, expression and recombination for the grouped data (Table 2).

### Effect of gene position on relative codon frequencies

Intragenic variation in codon use exists: optimal codons may for example increase along genes due to selection against non-sense errors [9,12]; also a decrease of GC-content along genes has been described [29,47]. To investigate an effect of gene position, I look at the four different measures of tables 1 and 2 for grouped amino acids:

F_OP_GC = frequency of optimal codons relative to all synonymous codons, but only for amino acids which have optimal codons that end with G or C;

F_OP_AT = frequency of optimal codons relative to all synonymous codons, but only for amino acids which have optimal codons that end with A or T;

F_GC_Opt = frequency of GC-ending optimal codons relative to all optimal codons; only for amino acids which have two optimal codons, one ending with G or C the other with A or T;

F_GC_Non-opt = frequency of GC-ending non-optimal codons relative to all non-optimal; only for amino acids which have at least two non-optimal codons, one ending with G or C and one with A or T.

Results show that optimal codons (F_OP_GC and F_OP_AT) tend to increase with gene position, and GC-ending ones (F_GC_Opt and F_GC_Non-opt) tend to decrease (Table 3). The result remains excluding the first 50 codons that often appear to be under different pressures [29,48]. The non-significant result for F_OP_GC may be due to conflicting pressures: optimal codons tend to increase while GC-ending codons tend to decrease. The non-significant result of F_GC_Opt may be due to dominant selection on optimal codons that is stronger than additional compositional forces.

**Table 3:** Effect of gene position.

	Position 1-300 (49-300)
**F**_**OP**_**AT** N = 27933 codons	+0.2679*** (+0.2656***)

**F**_**OP**_**GC** N = 33897 codons	+0.1244* (+0.0648NS)

**F**_**GC**_**Opt** N = 19794 codons	-0.0647NS (-0.1091NS)

**F**_**GC**_**Non-opt** N = 24446 codons	-0.1227* (-0.1591**)

Spearman Rank Analysis of frequencies of optimal and GC-ending codons with gene position for position numbers 1-300 and 49-300. *P < 0.05, **P < 0.01,***P < 0.001, NS = not significant.

An increase (or decrease) of certain codons along genes will as a side effect cause a positive (or negative) correlation of these codons with gene length [12]. To control for this effect of gene position, I take genes that are greater than 300 codons and only consider the level of bias in those codons from number 50 up to codon number 300 [12]. I repeat the two previous analyses (Tables 1 and 2) for the grouped data. The general patterns that GC-ending codons tend to decrease with gene length and increase with recombination remains, but becomes non-significant for the correlation between F_GC_Non-opt and gene length (Table 4). The positive correlation between F_OP_AT and gene length disappears indicating selection for translational accuracy may mainly act against non-sense errors [see [12]]. The two measures that showed no significant effect of gene position, F_OP_GC and F_GC_Opt (Table 3) remain significantly negative correlated after the control of gene position (Table 4). MR analysis of log-transformed variables indicates no independent effect of gene length for any codon class after controlling for position (Table 4). The results hence support that GC-ending codons tend to decrease along genes, and that this decrease along genes contributes considerably to the negative correlation of GC-ending codons with gene length.

**Table 4:** Controlling the effect of gene position.

	Gene length	Expression	Recombination
**F**_**OP**_**AT** N = 147193 codons R^2 ^= 0.260*** P(GL)NS,P(Expr)***, P(dmc1, mre11_0_)***	-0.0480NS	+0.3696***	Co:-0.1064*** nCo:-0.0922*** spo11:-0.0442NS dmc1:-0.1224*** mre11_0_:-0.0520* mre11_6_:-0.0922***

**F**_**OP**_**GC** N = 180789 codons R^2 ^= 0.445*** P(GL)NS, P(Expr)***, P(mre11_0_)*, P(dmc1)***	-0.1289***	+0.3839***	Co:+3284*** nCo:+0.2472*** spo11:+0.3909*** dmc1:+0.4829*** mre11_0_:+2648*** mre11_6_:+0.2669***

**F**_**GC**_**Opt** N = 70401 codons R^2 ^= 0.174*** P(GL)NS, P(Expr)NS P(dmc1, mre11_0_)***	-0.0751**	+0.1216***	Co:+0.2735*** nCo:+0.1843*** spo11:+0.3232*** dmc1:+0.3837*** mre11_0_:+0.2104*** mre11_6_:+0.2368***

**F**_**GC**_**Non-Opt** N = 126878 codons R^2 ^= 0.224*** P(GL)NS, P(Expr)*** P(dmc1, mre11_0_)***	-0.0481NS	+0.0410NS	Co:+0.2990*** nCo:+0.2339*** spo11:+0.3506*** dmc1:+0.4390*** mre11_0_:+0.2311*** mre11_6_:+0.2754***

Spearman Rank Correlations between frequencies of optimal and GC-ending codons with gene length, expression, and various recombination measures after controlling for a potential effect of gene position. The same 1571 genes were used for all measures. Presented are also MR results of variables whose log-transformation did not grossly deviate from a normal distribution. *P < 0.05, **P < 0.01,***P < 0.001

### Substitution rates and patterns

For both, GC-biased gene conversion as well as translational selection, we may not only expect relative codon frequencies to mirror the respective forces, but also substitution patterns. To disentangle the effect of gene conversion from translational selection, I take four-fold degenerated changes that do not change the non-optimal codon status and estimate four rates of substitutions AT->GC, GC->AT, AT->AT, and GC->GC. Under GC-biased gene conversion, we expect an increase of AT->GC (and a decrease of GC->AT) with recombination. As for the relative frequencies, the same genes are used for comparison, so a control of other variables should not be necessary to highlight their differences. However, while differences with respect to recombination can indeed be observed, a general decrease of all substitution rates with gene expression (and increase with gene length) confounds the observation (Table 5). Higher expressed (and shorter) genes appear more constraint even at non-optimal sites and indicate again beneficial roles of non-optimal codons for gene expression.

**Table 5:** Substitution patterns.

	Gene length	Expression	Recombination
**2761 genes**			

**AT -> GC** n = 47762 conserved, 7014 changes R^2 ^= 0.038*** P(GL)***,P(Expr)** P(dmc1)***,P(mre11_0_)NS	+0.1082*	-0.1922***	Co:+0.0178NS nCo:+0.0041NS spo11:+0.0218NS dmc1:+0.0290NS mre11_0_:+0.0252NS mre11_6_:+0.0287NS

**GC -> AT** n = 26739 conserved, 5677 changes R^2 ^= 0. 068*** P(GL)***,P(exp)** P(dmc1, mre11_0_)***	+0.2193***	-0.2435***	Co:-0.1277*** nCo:-0.0338** spo11:-0.1502*** dmc1:-0.1636*** mre0:-0.1193*** mre6:-0.1112***

**GC -> GC** n = 26739 conserved, 842 changes R^2 ^= 0.349*** P(GL)***,P(expr)NS, P(dmc1)***,P(mre11_0_)**	+0.2048***	-0.1615***	Co:+0.0210NS nCo: +0.0343NS spo: +0.0027NS dmc1:-0.0234NS mre0:+0.0173NS mre6:-0.0160NS

**AT -> AT** n = 47762 conserved, 281 changes R^2 ^= 0.746*** P(GL)***,P(expr)***, P(dmc1, mre11_0_)NS	+0.1559***	-0.0454NS	Co:-0.0156NS nCo:-0.0388* spo:-0.0804** dmc1:-0.0735*** mre11_0_:-0.0208NS mre11_6_:-0.0063NS

**AT->GC/(AT->GC+GC->AT)**	-0.0752***	+0.0441*	Co:+0.0983*** nCo:+0.0211 NS spo11: +0.1103*** dmc1:+0.1213*** mre11_0_:+0.0947*** mre11_6_:+0.0946***

**1965 genes; controlling for effects of gene position**			

**AT-> GC** n = 29013 conserved, 4126 changes R^2 ^= 0.011** P(GL)NS,P(expr)NS, P(dmc1, mre11_0_)*	-0.0281NS	-0.1210***	Co:+0.0264NS nCo:-0.0084NS spo11:+0.0241NS dmc1:+0.0418NS mre11_0_:+0.0077NS mre11_6_:+0.0416NS

**GC-> AT** n = 18164 conserved, 3594 changes R^2 ^= 0.055*** P(GL)NS,P(Expr)* P(dmc1, mre11_0_)***	+0.0118NS	-0.1420***	Co:-0.1025*** nCo:-0.0765*** spo11:-0.0848*** dmc1:-0.1117*** mre11_0_:-0.1004*** mre11_6_:-0.0704***

**GC-> GC** n = 18164 conserved, 509 changes	-0.0136NS	-0.0748***	Co:+0.0345NS nCo:+0.0143NS spo11:+0.0556* dmc1:+0.0433NS mre11_0_:+0.0038NS mre11_6_:+0.0214NS

**AT-> AT** n = 29013 conserved, 171 changes	-0.0460*	+0.0322NS	Co:-0.0151NS nCo:-0.0644** spo:-0.0648* dmc1:-0.0377NS mre11_0_:+0.0031NS mre11_6_:+0.0285NS

**AT->GC/(AT->GC+GC->AT)**	-0.0329NS	+0.0131NS	CO:+0.0824** nCo:+0.0642** spo11:+0.0664** dmc1:+0.0932*** mre11_0_:+0.0609** mre11_6_:+0.757**

Spearman Rank Correlations of directed substitutions at four-fold degenerated sites (that to not change the non-optimal status) and of the proportion of AT->GC substitutions with gene length, expression, and various recombination measures, with and without controlling for an effect of gene position. Presented are also MR results of variables whose log-transformation did not grossly deviate from a normal distribution. *P < 0.05, **P < 0.01,***P < 0.001, NS = not significant.

To look at the proportion of the rates of AT->GC substitutions from all AT <->GC substitutions will control for this general increase in substitution rates. According to Sueoka [49], one may estimate the equilibrium of GC content, GC*, to which a sequence is evolving to by GC* = u/(u+v), with u = rate AT->GC, and v = rate GC->AT. Sueoka's model assumes that all sites within a sequence evolve independently and as CpG do not appear hyper-mutable in yeast [50] one may use this simple approach. To control for translational selection, I again only consider four-fold degenerate changes that do not change the non-optimal codon status and estimate the proportion of AT->GC. This confirms an increase of the proportion of AT->GC substitutions with recombination as expected under GC-biased gene conversion and a decrease with gene length (Table 5). The proportion AT->GC further slightly increases with expression level (Table 5); this may be due to coupling of recombination and expression in yeast or indicate again a selective force favouring GC.

After control for gene position, the decrease with expression, and the opposing effects of recombination on rates of GC->AT and AT->GC remain (but the latter are not significant for the rate of AT->GC, Table 5). Interestingly, the general increase of substitution rates with gene length disappears. Also, the proportion of AT->GC changes does not correlate with gene length anymore, indicating again the effect of gene position. Why the rate of AT->AT changes decreases with gene length after control of gene position is unclear (Table 5).

MR analysis of log-transformed variables confirms independent effects of gene length, recombination and expression for AT->GC and GC->AT changes; independent effects of gene length disappear after controlling for gene position (Table 5). Only little of the overall variation for AT->GC and GC->AT changes is explained by the variables (Table 5).

### Recombination measures

The different recombination measures all tend to be conform in their general result; just for the substitution analysis, not all recombination measures support the finding. In general, dmc1 double-strand break (DSB) data tends to correlate strongest with GC-ending codons, followed by spo11 (spo) DSB and crossing-over (Co) events. But also non-crossing over events (nCo) and mre11 DSB data before and after meiosis and recombination (mre11_0 _and mre11_6 _respectively) confirms the finding. For the MR analysis, only dmc1 and mre11_0 _were considered as their log-transformation deviated the least from the bell-shaped normal distribution.

## Discussion

Separating translational selection from base composition indicates that in yeast frequency of GC-ending (optimal AND non-optimal) codons decreases with gene length and increases with recombination. This effect of base composition cannot be explained by variation in strength and efficiency of translational selection. GC-biased gene conversion appears the most likely explanation for the correlations with gene length and recombination. A decrease of GC-ending codons along genes is indicated and contributes to the decrease with gene length. Patterns of synonymous substitutions at four-fold degenerated sites support differences between AT->GC and GC->AT substitutions related to recombination, as expected under GC-biased gene conversion. Selection on GC-ending codons, for instance due to selection mRNA secondary structures may also contribute.

In models of homologous recombination that relate to double-strand break (DSB) repair, mismatches in the formed heteroduplex may be repaired by gene conversion, i.e. the conversion of one DNA strand into another, and the formed Holliday junction can then either be resolved with or without crossing-over [51-54]. Gene-conversion is biased towards -GC in yeast as in several other organisms and has been suggested before to affect synonymous codon use [see for review [36]]. However in studies investigating the effect of gene conversion on codon use or on patterns of divergence and polymorphism effects of gene length and gene position have not been considered before. An effect of gene position and length may arise as gene conversion events are not randomly distributed across the genome; the number of gene conversion events should for example relate to the number of initiating DSBs and interestingly the number of DSBs already decreases with gene length in the yeast data assembled here (-0.2590***). It is known that DSBs are often located in promoters with highest conversion numbers near the initiating DSBs [55-57]. Especially with short conversion tract lengths, the probability of gene conversion will hence decrease with distance from the DSB, i.e. from the promoter. If GC-biased, gene conversion could hence cause the negative correlation of GC-ending codons with gene position and thereby gene length. Gene conversion gradients, the decrease of gene conversion from one end of the gene to the other, have been observed in *S. cerevisiae *and other fungi [see [58] and references therein]. This decrease further seems often uni-directional in the 5'->3' direction, which may be due to various reasons including a gradient in heteroduplex formation or a gradient in the relative repair with gene conversion (as opposed to repair with restoration) [59,60].

Three recent studies in yeast are interesting with respect to the results of this study and GC- biased gene conversion. Noor [61] looks at the correlation of intergenic and intronic substitution rates with recombination to test for mutagenic effects of recombination on substitution rates; the lack of an increase of substitution rates with recombination however suggests mutagenic effects of recombination do not affect the substitution rates in yeast very much. The decrease Noor [61] observes instead is conform with GC-biased gene conversion; however surprisingly, he finds no difference between GC->AT and AT->GC substitutions associated with DSBs. Weber and Hurst [62] find a decrease of non-synonymous substitution rates with recombination, and interestingly not only with crossover but also with non-crossover events, which again is conform with GC-biased gene conversion. Referring to Noor's [61] lack of difference between GC->AT and AT->GC, they do not discuss GC-biased gene conversion further. Harrisson and Charlesworth [63] investigate the effect of GC-biased gene conversion in much detail, but do not consider the negative correlation between optimal codons and gene position and length. The potential contribution and importance of GC-biased gene conversion to observed patterns of substitutions for primates and humans have been highlighted much recently [see e.g. [64-67]] and in humans gene conversion tracts are short and steep [68]. It would be interesting whether an effect of gene position and length may be observed.

Besides a compositional bias in repair, such as GC-biased gene conversion, a negative correlation of GC-content at synonymous sites with gene length could also be due to a compositional bias in selection or mutation. Selection alternative to translational selection may differentiate among GC- and AT-ending codons, and its strength or efficiency could correlate with gene length and position. One selective target related to gene length and GC-content is the stability of secondary structures. Thermodynamic stability of mRNA structures increases with gene length and GC-content as the absolute thermodynamic stability of a sequence will depend on the absolute number paired bases, and their strength of bonds, which is highest for pairs of G and C. Laboratory studies suggest too stable secondary structures within the protein coding part interfere with translation [69], in which case selection should dis-favour too stable structures. Especially in genes that are more stable through their length, lower level of GC may be beneficial and selected for. This could theoretically lead to the observed negative correlation of GC-ending codons with gene length. It may also explain a negative correlation of the GC-content with expression level (Table 2): first, selection on thermodynamic stability may vary with expression, and secondly, higher expressed genes will be shorter, and if the absolute stability would be under selection, their GC-content may be higher. Whether selection favours or dis-favours stability in protein coding regions however is a controversial issue [see e.g. [43-46]], for yeast, selection may indeed act against too stable structures [46]. However, even under selection against too stable secondary structures in coding mRNAs, it is not obvious why there should be a decrease of GC with gene position.

Variation in rate or bias of mutations may also contribute to the observed patterns, and mutations appears to be biased towards AT in yeast [70]. In this case however, polymorphism data should show the same patterns as divergence.

Note that in contrast to yeast, in *E. coli*, relative optimal codon use of all amino acids increases in frequency with gene length; this is independent of whether the optimal codon ends in AT- or GC- [12]. A possibly related difference between prokaryotes and eukaryotes is indicated in a separate line of studies: in prokaryotes GC-content increases with gene length [71,72], while in eukaryotes it mainly decreases [72]. It will be interesting to check other organisms for a decrease of GC-ending codons with gene length and gene position, and evaluate the effect of base composition - and GC-biased gene conversion - on synonymous (and optimal) codon use and sequence evolution.

GC-biased gene conversion may contribute to the negative correlation of (GC-ending) optimal codons with gene length described in various organisms, which so far has been explained only by variation in the strength or efficacy of translational selection. Hill-Robertson Interference has been suggested to cause the negative correlation between optimal codon use and gene length, and while Hill-Robertson Interference may contribute to several patterns of synonymous codon use [e.g. [16,23-29]], it cannot easily explain the here described compositional correlations for yeast that affect both optimal and non-optimal codons. Loewe and Charlesworth [27] included gene conversion in their model of intragenic background selection and highlight its contribution in breaking down linkage. It will be interesting to set up models that include also current knowledge on gene conversion bias towards GC, distribution of DSBs, e.g. the relation to promoters, the conversion lengths and dependencies on homology.

## Conclusion

Separating translational selection from base composition indicates that in yeast frequencies of GC-ending (optimal AND non-optimal) codons decrease with gene length and position and increase with recombination. GC-biased gene conversion appears the most likely explanation. Substitution patterns support effects of GC-biased gene conversion. These results are of interest for our understanding of the process of gene conversion and its implications, but also for interpreting the negative correlation between optimal codon use and gene length observed in various organisms whose optimal codons tend to end with -GC.

## Methods

### Data

I used the data set kindly provided by Weber and Hurst [62]. This data includes (i) alignments of *S. cerevisiae*, *S. mikitae *and *S. paradoxus*, (ii) expression data [73], (iii) crossover and non-crossover recombination events [74], Spo11 double-strand break (DSB) data [56], Dmc1 DSB data [75], and Mre11 DSB data preceding meiosis and recombination and Mre11 DSB data after recombination, mre11_0_and mre11_6 _respectively [76].

### Optimal Codons

Optimal codons are defined as in [77] 12 optimal codons end with G or C (-GC), 12 with A or T (-AT), 17 non-optimal ones end with -GC, 18 with -AT. Throughout the paper, the terms "optimal" and "non-optimal" will refer to translational selection alone. Codon identification is based on the *S. cerevisiae *sequence.

### Difference between optimal codons depending on whether they end with -AT or -GC

To check whether the correlation between the relative frequency of optimal codons and gene length differs for AT- and GC-ending optimal codons, I compute F_OPi_, the relative frequency of optimal codons (F_OP_) for each contributing amino acid (i) separately: F_OPi_, = number of respective optimal codon divided by the number of all codons for the respective amino acid. For amino acids with both - one AT- as well as one GC-ending optimal codon (Thr, Val, Ile, Ser), I compute the relative optimal codon frequencies of the two optimal codons separately. Serine for example has two optimal codons, TCT and TCC, and if I would count one TCT, two TCC and five non-optimal codons in a gene, F_OP_TCT = 1/(5+1) and F_OP_TCC = 2/(5+2). I further group amino acids with AT- (Ala, Arg, Gly, Gln, Glu, Pro, Cys) and GC-ending (Leu, Lys, Phe, Tyr, His, Asp, Asn) optimal codons and compute the relative frequency of optimal codons across them.

### Effect of base composition independent of translational selection

For amino acids with at least two optimal (Ile, Ser, Thr, Val) or two non-optimal codons (Ser, Thr, Val, Pro, Ala, Arg, Gly, Leu), one can further control for effects of translational selection by separately computing the relative frequencies of GC-ending optimal (F_GC_optimal) and non-optimal (F_GC_non-optimal) codons. For example, if I would count 15 serine codons in a gene, 9 of which are optimal and three of the nine optimal codons end with -GC, then F_GC_optimal = 3/9; if 2 of the remaining 6 non-optimal Serine codons end with -GC, F_GC_non-optimal = 2/6. Again, I also group the optimal or non-optimal codons of the respective amino acids and compute relative frequencies of GC-ending codons across them.

### Effect of gene position on relative codon frequencies

Codon use may vary along genes, e.g. optimal or GC-ending codons may increase or decrease along the length of a gene [29,40]. Such an increase or decrease with gene position is of interest for itself, but also, as it can affect the correlation with gene length. To investigate the effect of gene position itself, I generate super-sequences for codon position 50-200 across genes [see [29]]. To control for an effect of gene position, I take genes that are greater than 300 codons and only consider the level of bias in those codons up to that length [12]. I exclude the first 50 codons that may be under conflicting selection pressures [29,76].

### Substitution rates and patterns

To investigate whether the substitution patterns supports GC-biased gene conversion, I look at all non-optimal four-fold degenerated sites (Pro, Thr, Val, Ala, Gly, Leu, Ser, Arg) that are conserved in amino acid as across the three yeasts. To control for translational selection, I only look at sites that are conserved in their non-optimal status across the three yeasts. I count sites with conserved codons, and sites with synonymous changes at the 3^rd ^codon position between *S. cerevisiae *and *S. mikitae *for which *S. mikitae *and *S. paradoxus *are conserved, and the change hence likely occurred in *S. cerevisiae*. I count substitution types (i) AT->GC, (ii) GC->AT, and (iii) AT->AT or (iv) GC->GC, and take their proportions relative to the respective codons that are conserved in *S. cerevisiae*, e.g. AT->GC/ATconserved. Also, I compare the rate of substitutions AT->GC to all AT <->GC substitutions.

### Statistics

I use Spearman rank correlation analyses to investigate the correlation of relative codon frequencies or the substitution rate estimates with expression, recombination, gene length and gene position. I further performed multiple regression (MR) analysis. While the log-transformed variables do not seem to deviate grossly from normal distribution, normality could not be established for the variables using Kolmogorov-Smirnov-Lilliefors test. It is known however that with large sample size, minor deviations from normality can be statistically significant. For recombination estimates, only dmc1 and mre11_0 _were used as they deviate the least from normality. In general, first order interaction terms did not increase the explainable variance significantly.

## Authors' contributions

NS carried out the analyses, conceived of the study, and wrote the manuscript.
